# 

**Published:** 1918-03-23

**Authors:** 


Rates of Subscription: Twelve months, 6/6; Six months, 4/-; three months, 2/-, post free to any address in the United Kingdom. Abroad, Twelve months, 8/8; Six months, 5/-; Three months, 2/6, post free. THE HOSPITAL "Index" to Volume LXII, April 1917 to September 1917, is now ready, price 2½d. per copy, post free. Address The Manager, The Hospital, "The Hospital" Building, 28/29 Southampton Street, Strand, London, W.C. 2.

### King Edward's Hospital Fund for London.

WE are asked to state that hospitals in the County of London, or within nine miles of Charing Cross, desiring to participate in the grants made by this Fund for the year 1918 must make application before March 31 to the Hon. Secretaries, 7 Walbrook, E.C. 4. Applications will also be considered from convalescent homes which are situated within the above boundaries, or which, being situated outside, take a large proportion of patients from London. Applications will also be considered from sanatoria for consumption which take patients from London, or which are prepared to place beds at the disposal of the Fund for the use of patients from London hospitals.

### Matrons' and Sisters' Appointments.

Ebbw Vale Workmen's Surgical Hospital.—Miss Ellen Lloyd has been appointed matron. She was trained at the Royal Free Hospital, has been sister at Watford Cottage Hospital, at Swansea General and Eye Hospital, and at the City of London Hospital for Diseases of the Chest, Victoria Park, and night superintendent at the Children's Infirmary, Liverpool. She has also done military nursing in France and at home.

Infants' Hospital, Vincent Square, Westminster.—Miss E. D. Townsend has been appointed ward sister. She was trained at Guy's Hospital and at the Brighton Hospital for Women and Children, and has been staff nurse at the East London Hospital, Shadwell.

Jessop Hospital for Women, Sheffield.—Miss Theresa Farley has been appointed ward sister. She was trained at the Essex County Hospital, Colchester, has been sister at the Coventry and Warwickshire Hospital, Hertford British Hospital, Paris, and at the Mansfield and District Hospital. She has also done military work.

Royal Albert Edward Infirmary, Wigan.—Miss Marian Newton has been appointed sister. She was trained at the General Infirmary, Burton-on-Trent, later being ward sister there, and has been sister at Tankerton Military Hospital, Whitstable.

Manor (County of London) War Hospital, Epsom.—Miss Alice Hayes has been appointed assistant matron. She was trained at the Township of South Manchester Hospitals, has been ward sister there and at Highfield Infirmary, Liverpool, night superintendent at Brighton Infirmary, and home sister and second assistant matron at St. James' Infirmary, Balham.

The Infirmary, Stockport.—Miss Jean Wightman has been appointed sister. She was trained at the North Lonsdale Hospital, Barrow-in-Furness, has been theatre sister at the Royal Buckinghamshire Hospital, Aylesbury, sister at the Deaconess Hospital, Edinburgh, and night sister at the Royal Infirmary, Perth.

V.A.D. Hospital, Buxton.—Miss Frances Corneille has been appointed matron. She was trained at the Edinburgh Royal Infirmary, and has held several appointments under Queen Victoria's Jubilee Institute. She has also been nursing in Canada, and with the British Red Cross Society.

War Hospital, Chester.—Miss J. G. Punshon has been appointed ward sister. She was trained at the Edmonton Infirmary, later being theatre sister there, and has been sister at the Merthyr Tydvil General Hospital.

**NOTE.—Particulars of Appointments for insertion in this column will be welcomed.**

